# Maintaining Cognitive Functioning in Healthy Seniors with a Technology-Based Foreign Language Program: A Pilot Feasibility Study

**DOI:** 10.3389/fnagi.2017.00042

**Published:** 2017-03-01

**Authors:** Caitlin Ware, Souad Damnee, Leila Djabelkhir, Victoria Cristancho, Ya-Huei Wu, Judith Benovici, Maribel Pino, Anne-Sophie Rigaud

**Affiliations:** ^1^Department of Psychoanalytical Studies, University of Paris VII DiderotParis, France; ^2^Department of Clinical Gerontology, Broca HospitalParis, France; ^3^Faculty of Medicine, University of Paris DescartesParis, France

**Keywords:** older adults, foreign language learning, technology, cognitive plasticity, cognitive reserve

## Abstract

Researchers have hypothesized that learning a foreign language could be beneficial for seniors, as language learning requires the use of extensive neural networks. We developed and qualitatively evaluated an English training program for older French adults; our principal objective was to determine whether a program integrating technology is feasible for this population. We conducted a 4-month pilot study (16, 2-h sessions) with 14 French participants, (nine women, five men, average age 75). Questionnaires were administered pre- and post-intervention to measure cognitive level and subjective feelings of loneliness or social isolation; however, these scores did not improve significantly. Post-intervention, semi-directive interviews were carried out with participants, and a content/theme analysis was performed. Five main themes were identified from the interviews: Associations with school, attitudes toward English, motivation for learning English, attitudes toward the program’s organization, and social ties. The program was found to be feasible for this age group, yet perceived as quite difficult for participants who lacked experience with English. Nonetheless, most participants found the program to be stimulating and enjoyable. We discuss different suggestions for future programs and future directions for foreign-language learning as a therapeutic and cognitive intervention.

## Introduction

As the population ages, more older adults will be at risk of developing neurodegenerative syndromes such as Alzheimer’s disease and other age-related dementias, yet no pharmaceutical treatment has been found to successfully prevent or delay the development of these neurological conditions (Gauthier et al., 2012; Selkoe, 2012). In the absence of pharmacological solutions, alternative interventions demand exploration (Hughes, 2010; Dresler et al., 2013; Law et al., 2014). Thus, there is significant interest in prevention studies that explore the effects of cognitive stimulation and/or physical exercise on the cognitive functioning of older adults. Along with age, genetics, level of physical activity, and subjective cognitive complaints, formal education level has been identified as an influential factor in the development of dementia (Kivipelto et al., 2006). In addition, both depression and loneliness have been shown to negatively affect global cognitive functioning (Tzang et al., 2015; Zhong et al., 2016), and involvement in social and leisure activities has been associated with a decreased risk of dementia in the elderly (Wang et al., 2002). Due to these findings, educational and social programs geared toward older adults, like *Senior Odyssey*, have been developed with the goal of maintaining healthy cognitive functioning in this population (Stine-Morrow et al., 2007). We propose focusing on a specific type of educational intervention involving second-language learning. To the authors’ knowledge, no foreign language-oriented program has been developed for cognitively healthy older adults with the goal of maintaining cognitive functioning.

In gerontology research, language is a significant field of study as the development of cognitive impairment or dementia can greatly affect its use (Forbes-McKay and Venneri, 2005). Furthermore, linguistic ability may contribute to cognitive reserve, the brain’s cognitive capacities that allow it to compensate for age or disease-related brain pathology (Gold et al., 2013), also defined as ‘functional resistance to structural brain damage’ (SantaCruz et al., 2011). Bilingualism has been shown to affect the brain’s anatomy, with lifelong bilinguals possessing greater white matter in the frontal lobes than their monolingual counterparts (Olsen et al., 2015). Bilingualism seems to play an important role in cognitive reserve, and thus may help to delay the onset of Alzheimer’s (Bialystok et al., 2007). Additionally, novelty and learning have been associated with the maintenence of cognitive plasticity in older adults (Greenwood and Parasuraman, 2010). In this vein, Antoniou et al. (2013) put forth the hypothesis that learning a foreign language could increase cognitive reserve in older adults. They suggest that learning a foreign language could improve cognitive plasticity as learning languages requires the utilization of extensive neural networks, soliciting working memory, inductive reasoning, sound discrimination, speech segmentation, task switching, rule learning, and semantic memory. Moreover, they posit that learning a foreign language could have important social implications, as gaining access to a second language could facilitate communication with foreigners and increase both travel and job opportunities. The development of foreign-language learning programs is needed to test these hypotheses.

Despite the common belief that seniors cannot learn foreign languages as successfully as younger adults or children, it has been shown that older adults do indeed have the capacity to learn a second language (Gómez, 2016). Moreover, seniors can relearn previously acquired words just as well as younger subjects (Van der Hoeven and de Bot, 2012), even years after the language was originally learned. Bahrick (1984) found that most second-language knowledge is retained for over 50 years, even without its use. However, learning a completely new language can be more challenging for older adults, as working memory is often impaired with age (De Bot and Makoni, 2006).

Naturally, seniors have special needs and interests concerning learning. In a study by Duay and Bryan (2008), the authors interviewed 36 older learners and identified three main determinants that facilitate effective learning: involvement, the instructor, and relevant, familiar topics. Seniors expressed a preference for taking an active role in learning through questions and discussion, and for flexible, organized instructors who taught with authority. Regarding setting, researchers have found that seniors prefer learning situations outside of the traditional classroom with classmates of similar age (Clough, 1992). Additionally, it has been shown that educational settings involving social activities and online programs increase older adults’ motivation to learn (Chang and Lin, 2011).

Recent research on computer-assisted language learning (CALL) suggests that technology could be an effective tool in learning a foreign language. Golonka et al. (2014) affirm that ‘technological innovations can increase learner interest and motivation; provide students with increased access to target language (TL) input, interaction opportunities, and feedback; and provide instructors with an efficient means for organizing course content and interacting with multiple students.’ In addition, the use of technology in language learning has been associated with autonomous learning (Godwin, 2015).

Computer-assisted language learning programs are varied and can include videos, online dictionaries (Ranalli, 2013), and games. Certain programs can be found online and accessed free of charge such as ‘Duolingo,’ ‘BBC Learning English,’ and ‘News in Levels.’ Youtube videos can also be utilized for foreign language learning (Terantino, 2011). Moreover, foreign-language vocabulary can be learned through viewing television series in the TL. In a study by Kuppens (2010), scores on English aptitude tests were higher for Belgian students who watched captioned English-language movies and television than for those who did not.

However, research has shown that older individuals do not use contemporary technologies as frequently as their younger counterparts, and that they are fairly resistant to embracing them (Purdie and Boulton-Lewis, 2003; Wu et al., 2015). Nevertheless, being computer savvy is not essential for participants to obtain positive results from technologically assisted learning (Kueider et al., 2012). The act of learning how to use a computer can add to a sense of mastery for aging subjects (Lee et al., 2013), and affect older adults’ well-being, sense of independence, and social relations (Shapira et al., 2007). Moreover, with the Internet as a resource for learning, individuals can find material that is of specific interest to them.

Inspired by Antoniou et al.’s (2013) article on the potential cognitive and social benefits of teaching a foreign language to older persons, we developed a foreign-language learning program geared toward French seniors that incorporated tablet computers. Our main objective was to examine the participants’ subjective experience of learning a foreign language in order to explore the feasibility of the program.

## Materials and Methods

### Participants

Participants were recruited from a panel of volunteers who had previously agreed to participate in research studies led at Broca Hospital, a Paris-based geriatric hospital. In total 14 (five men and nine women) mean age 75, community-dwelling, cognitively healthy older adults without auditory or visual impairment were recruited. Baseline cognitive level was determined with Nasreddine et al.’s (2005) French version of the Montreal Cognitive Assessment (MoCA). Most participants had completed over 12 years of formal education, and thus had some exposure to the English language. Level of English proficiency was determined by the instructor during the first meeting through informal conversation and questions concerning the participants’ previous experience with English. Five participants were identified as beginner, five as intermediate, and four as advanced. **Table 1** presents participant characteristics. All participants signed consent forms to participate in this experiment, and the study was approved by the local Committee of Ethical Evaluation for Research in Health.

**Table 1:** Participant demographics.

Characteristics	Participants (*n* = 14)
Age	75.42 (±8.93)
Mini/Maxi	63/90
Male/Female	5/9
Education	5.1 (±1.4)
Beginner English level	5
Intermediate English level	5
Advanced English level	4
MOCA	26 (±2.67)

This qualitative and exploratory study was structured into two phases: The development and design of the program with an interdisciplinary team of professionals involved in geriatric care, and program implementation and assessment with participants. The research was conducted at LUSAGE Laboratory at Broca Hospital, between April and July of 2014.

### Measures

In addition to semi-structured qualitative interviews, two quantitative measures were included. The MoCA is a brief cognitive test designed to efficiently evaluate global cognitive functioning in older adults. Russell’s (1996) University of California Loneliness Assessment (UCLA) scale is designed to measure subjective feelings of loneliness or social isolation in youth, adults, and older adults. In our study, we used Grâce et al.’s (1993) French version of the UCLA.

### Development and Design of the Program

While we initially envisaged an onsite learning program in a group setting with an instructor, we then considered using technology to facilitate offsite learning in order to increase the program’s intensity. We therefore decided to develop a program with a multimedia approach using online videos and dictionaries on tablets or PCs. Participants who owned a laptop or tablet would be encouraged to train at home by connecting to the websites shown in class in addition to attending the group lessons.

With the intention of catering to participants’ interests and reviving memories from their youth, we selected content from public-domain television series that aired in the 1950s and 1960s, as well as popular music by artists of the era available online. Not only have songs been proven to be an effective way of teaching a foreign language (Ludke et al., 2014), we hypothesized that using this material might be a more enjoyable and stimulating approach than traditional lessons involving written texts or grammar textbooks. Moreover, familiar topics have been shown to be better understood in a foreign language than unfamiliar topics (Schmidt-Rinehart, 1994). Using series that may be familiar to participants might increase their interest and motivation to understand the content. Based on the contents of each scene or song, we structured each lesson around a specific theme, as shown in **Table 2**.

**Table 2:** Themes of the sessions.

Lesson 1: Meeting for the first time	Lesson 9: Blind date
Lesson 2: Making breakfast	Lesson 10: Getting ready to go out
Lesson 3: Gardening	Lesson 11: Antiques and bargaining
Lesson 4: Dinner with colleagues	Lesson 12: Sleeping and waking up
Lesson 5: Shopping for clothes	Lesson 13: Getting hurt
Lesson 6: Taking care of pets	Lesson 14: Expressing nostalgia
Lesson 7: Expressing love	Lesson 15: Leaving for a trip
Lesson 8: Jealousy and trust	Lesson 16: Newspaper and learning websites

### Implementation of the Program

The program encompassed sixteen 2-h group sessions with 16 topics, held once a week over a 4-month period.

For each session, tablets and a transcription of a scene in English were distributed to the participants. Each line was enumerated, and the participants were asked one at a time to read a line out loud and to attempt to translate that line. The participants were encouraged to use their tablets to look up any unknown words on an online dictionary. Afterward each of these words’ translations were written on the board by the instructor. The participants wrote the translation of each line on their copy of the transcription. Once the scene was translated, each participant was asked again to individually read a line out loud. The scene was then shown again, this time with the subtitles in French. After each line, the scene was paused in order for the group to repeat the line out loud in unison. After the scene, a time for questions was allowed. Finally, English-learning websites, as well as Youtube music videos were shown to the participants in order to familiarize them with learning methods that could be continued at home.

All sessions were conducted by a native English-speaking psychologist with experience teaching English to adults and children. Two other French-speaking psychologists attended the sessions to facilitate use of the tablets and laptops.

### Assessment of the Program

At pre- and post-intervention, the French versions of the MoCA and the UCLA were administered. Following the end of the 4-month training period, the participants met individually with one of the psychologists for an interview concerning his or her experience in the group. The semi-structured interview included, but was not limited to, these five questions:

(1) What was your experience in the group?
(2) What are your suggestions?
(3) What did you think of the group dynamic?
(4) What did you think of learning English with the tablets?
(5) Do you have anything else to add?

### Quantitative Analysis

Pre- and post-intervention scores of the UCLA and the MoCA were analyzed with a paired sample *T-*test.

### Qualitative Analysis

The interviews were recorded and transcribed. A content/theme analysis using the method proposed by Braun and Clarke (2006) was performed. After careful word-by-word transcription, the interviews were read separately multiple times by three researchers. Each researcher coded every transcript separately, and the ideas expressed in the interviews were regrouped into themes. The themes were then analyzed by the psychologist team. Afterward, the interviews were read again to check the accuracy of the themes. The quotes selected from the participants’ interviews were translated from French to English by the first author.

## Results

Ten participants attended the sessions regularly and were interviewed at the end of the program. Four participants attended the sessions sporadically and were not available for the final interviews, three of whom were beginners in English.

Paired sample *T-*test of pre- and post-intervention data for the UCLA and the MoCA showed no significant change in scores. These results are shown in **Table 3**.

**Table 3:** Pre–post intervention results.

Scale	Pre	Post	*P*
MOCA	26.46	26.77	0.67
UCLA	39.15	40.83	0.15

Five main themes were identified in the qualitative analysis of the interviews: associations with school, attitudes toward learning English, motivation for learning English, attitudes toward the program’s organization, and social ties. These themes are detailed in **Table 4**.

**Table 4:** Main themes and subthemes.

Main themes	Subthemes and number of participants concerned
Associations with school	- School memories (10) - Comparing the program with school (3)
Attitudes toward English	- Learning English is fun (7) - English is difficult to understand (6) - Learning English is beneficial (4) - Learning English is essential (3)
Motivation for learning English	- Connecting with family (3) - Desire for more (3) - Getting out of the house (5)
Attitudes toward the program’s organization	- Different English levels are challenging (9) - Technology and English learning are linked (5) - Immersion is needed (3) - It went too quickly, feeling left behind (3) - Focus on spoken English (2) - Scenes from the series (3)
Social ties	- Absence of bonding (10) - Convivial atmosphere (9) - ‘Our generation’ (4)

### Theme 1: Associations with School

All of the participants mentioned school during the interviews, whether it was in comparison to the program or in reminiscing about their first experiences with English. In particular, one participant spoke about how learning English brought back memories: ‘There’s an advantage to re-sourcing, to coming back to one’s sources […] coming back to former memories is very good’ (P1, male 70). Another participant said that the experience made him feel younger. ‘I really liked it, it made me, well, it made me feel a bit younger’ (P2, male 73).

Some participants mentioned that the group was beneficial specifically because it differed from their school experience:

Doing common, current English, I found that to be very positive, because it completes official education. I felt the benefits because, as I told you, she familiarized us with usual daily expressions or journalistic expressions that we don’t learn (usually) (P4, male 87).

However, sometimes the association with school seemed to activate feelings of inadequate education, ‘I only completed middle school, and when I passed the last year I only had two over 20 in English, so I was never able to catch up’ (P5, female 67).

### Theme 2: Attitudes toward Learning English

#### ‘English Is Difficult’

Most of the participants expressed that English was difficult to understand, and that new words were hard to remember: ‘I remember words that I learned 40 years ago, but I must repeat and repeat the words that I learn nowadays and it’s annoying’ (P7 female 74).

Three participants expressed how English was overwhelming to them, and that they felt left behind in the program. However, they also mentioned that their comprehension improved as they continued.

‘I was a little overwhelmed by the events, you see? Especially concerning English. I said to myself that I was behind. And then I wasn’t traumatized because we were all pretty much the same level and in the same category’ (P7 female 74).

#### Stimulating and Fun

Most participants said that the sessions were amusing, playful, and fun. The group seemed to enjoy the sessions; many said they were a pleasure to attend. Other participants mentioned the stimulating and mind-opening quality of the group: ‘it’s very good for memory, to speak it effectively, it obliges certain brain zones to work,’ (P8, male 66) and ‘it opens up your mind, it’s better than staying home and doing nothing … I like to participate so it’s pretty interesting to discover something else actually’ (P5 female 67).

#### English Is Essential

Some emphasized the importance of the English language. One participant commented, ‘First of all, English has become *indispensable*, we are integrated into the English language’ (P1, male 70). Another expressed, ‘English is the most common, the most necessary in daily life, the most current’ (P3, female 90). Similarly, one participant noted that speaking a second language is profitable: ‘It’s an advantage in the work world to be able to master a second language, it’s a *richness*’ (P9 female 68).

### Theme 3: Motivations for Learning English

#### Desire for More

Participants expressed a desire for a more expansive English-language program. In particular, participants mentioned wanting a wider range of subjects to learn, more locations for the groups to gather, and more volunteer educators: ‘The question would be to spread out this type of activity. There will be more and more elderly, it would be good to have more and more activities like this’ (P4, male 87). ‘I would like to do the same thing in German, and even in Spanish…’ (P3, female 90).

Some expressed a desire to continue the English group, asking when the sessions would recommence. One participant expressed that, although she did not have time to pursue English, she would remember the English-learning websites for the future. She insisted:

I’ll remember, I’ll remember, I’ll remember, and I’m keeping it handy, and I won’t forget, and I think that when I have more personal time, I’ll put myself to it (P9, female 68).

#### Family

Three participants specifically mentioned family living abroad when discussing English. In particular, when asked if she would continue learning English in the future, one participant said that she is now less motivated because her daughter has moved back to France: ‘I would like to, but I’m less motivated, you know why, my daughter is here, and she will not go back to the United States. It motivated me, you understand’ (P7 female 74).

#### ‘Getting Out of the House’

Finally, it seems that the idea of getting out of the house was an important motivating factor for participation in this study. One participant expressed thusly, ‘first of all, it got me out of my place because I had a goal, and it brought me a lot, I found that it was very nice’ (P7, female 74).

### Theme 4: Attitudes toward the Program’s Organization

#### Different Levels in English

The participants’ different levels in English proficiency were mentioned consistently: ‘I found that there were people who knew too much, I found that they didn’t really belong in the group’ (P5, female 67). Another participant shared his feelings of inadequacy in comparison to the other participants, saying that he felt uncomfortable because he didn’t know as much as the others did, and that his English level was quite poor in comparison (P2, male 73). Alternatively, one of the participants emphasized the fact that the different proficiency levels in English and technology facilitated contact with others as she had to ask others for help (P10, female 63).

#### Technology

Another prevalent topic the participants addressed was the use of technology. Opinions were divided between those who found that it was helpful to use the tablets in learning English, and those that considered it to be useless or overly time-consuming. One participant said that learning English helped her to learn how to use the tablets, ‘firstly it allowed me to learn about the tablets by looking up words even though it made the translation take longer’ (P7, female 74). For some, the use of the tablets seemed to stimulate motivation to learn English. One participant expressed; ‘It’s really very amusing, it’s stimulating, it motivates you to make progress, to look up alphabets, dictionaries, I found it very good’ (P3, female 90). Another participant mentioned a link between learning English and technology: ‘There’s a connection, and it allowed me to discover your dictionary with many meanings’ (P7, female 74).

#### Scenes of the Series

Three participants mentioned the videos viewed during the sessions. One beginner participant commented that she was not always able to understand everything just by watching the video. Another participant remembered watching one of the series in French as a child, and therefore said she very much enjoyed rediscovering it in English. One of the participants purchased a DVD of one of the series used during the lessons for his spouse who has Alzheimer’s.

#### Rhythm

The rhythm of the class was an issue for some participants. Three participants said that they felt lost at times during the sessions:

It was a bit fast, to see, and to write, and to understand … to translate lots of new words, expressions and everything, it was a bit dense … there were so many things that I became as if I was little bit of a beginner again (P10, female 63).

#### Immersion

Some participants emphasized the importance of immersion. Three participants said that if they had been immersed in the language they would have learned more. In this vein, one participant suggested that linguistic trips could be organized for the elderly, just as they are often organized for youth (P8, male 66).

#### Use of Spoken English

The oral use of English was seen as beneficial. One beginner-level participant mentioned that saying things out loud made her learning experience more enjoyable: “After I felt much more comfortable, the fact that you had us first say things out loud, even if I didn’t understand anything, it wasn’t a big deal (P5, female 67). Another participant mentioned how learning to speak English was more important than learning to read it, as most of them had already learned to read English at school (P8, male 66).

### Theme 5: Social Ties

When asked about the group dynamic, all of the participants expressed that they did not establish strong social ties with one another. This did not seem to concern everyone, but two participants in particular regretted this lack of bonding: ‘What I regret is that we weren’t able to establish a connection, I mean in order to keep in touch, even through the internet, I didn’t find it…everyone has their own life’ (P7 female 74). Another participant commented: ‘I would have really liked to spend a little time on the phone with my colleagues … I regret it a little, because we could have had more of a relationship’ (P2 male 73).

Nonetheless, almost all the participants remarked on the convivial atmosphere of the group, calling it ‘sympathique,’ a common word in French that can be translated as ‘nice, pleasant, or enjoyable.’ Four participants used the phrase ‘our generation’ during the interviews. In particular, one participant mentioned that people of their generation do not make friends easily, that although they can make acquaintances, building friendship is much more difficult at a later age (P8, male, 66).

## Discussion

The objectives of this study were to develop a technology-based, foreign-language intervention geared toward seniors and to evaluate its feasibility. The qualitative data suggest that this intervention, although perceived as difficult by some, is feasible for older adults. The main themes expressed in the interviews with participants were organized into five categories: associations with school, attitudes toward English, motivation for learning English, attitudes toward the program’s organization, and social ties. Although cognitive and loneliness perception scores did not significantly improve in our study, we discuss the potential social and cognitive benefits of foreign-language learning interventions for older adults.

### School Memories

During the interviews about their experiences in the program, all of the participants mentioned school, either reminiscing about their education and youth or comparing the current intervention to their past experience with English. For some, this association was positive: ‘Learning English brings back old memories, it’s good to go back to one’s sources.’ This could be compared to reminiscence therapy, in which reactivating past memories could improve self-acceptance, resolve past conflicts, and provide perspective (Gaggioli et al., 2014).

### English Is Difficult, Fun, and Essential

As shown in the qualitative results, English was perceived to be difficult, fun, and essential. Participants who had limited prior experience with English seemed to be the most challenged, saying that English was difficult to understand. Even some of those who had experience with English expressed difficulty retaining new vocabulary, which seemed to cause frustration. Undoubtedly, short-term memory deficits constitute an added roadblock for older learners (De Bot and Makoni, 2006).

Nonetheless, the difficulty of language learning could determine its cognitive impact. Schroeder and Marian (2016) claim that improvements in cognitive processing occur with repetitive and challenging activities due to the ‘supply demand mismatch’ hypothesis. The authors explain that when cognitive resources do not meet cognitive demands, cognitive supply is increased. The challenge of foreign-language learning could therefore determine the extent of its cognitive benefits. With repetitive practice, cognitive capacities expand to meet cognitive demands. Indeed, participants who had expressed difficulty learning English at the start of the program said that it eventually became easier. In addition, some participants said that learning English was ‘fun,’ ‘stimulating,’ and ‘mind-opening.’ This coincides with the finding that cognitive training has been shown to affect older adults’ openness to experience (Jackson et al., 2012). Learning a new language could similarly affect an individual’s open-mindedness. Other participants described English as ‘essential,’ ‘indispensable,’ or ‘profitable.’ These perceptions could have contributed to participants’ motivation for learning English.

### Motivations for Learning English

Learner motivation plays an important role in language acquisition. It can compensate for deficiencies in foreign-language learning aptitude and even weaknesses in the course’s methodology (Marinova-Todd et al., 2000; Dörnyei, 2005). Kim and Kim (2015) found that ‘self-actualization,’ defined as ‘the feelings of satisfaction and delight … from knowing English’ to be the most powerful motivating factor among Korean learners of English.

With sufficient personal motivation, learning a foreign language is feasible for older adults. Participants in our study expressed their motivations for learning English in terms of their desire to continue the program, family connections, and opportunity to ‘get out of the house.’ Variations of this last theme have been observed in other studies on older adult learning. According to Duay and Bryan (2008), 66% of seniors interviewed said that their engagement in educational activities allowed them to stay involved with the outer world. In turn, it seems that learning activities could help older adults maintain a connection with society and their peers, while remaining mentally, physically, and socially active (Purdie and Boulton-Lewis, 2003).

### English Level Heterogeneity and Technology

Participants commented most on the heterogeneity of English proficiency levels, the rhythm of the sessions, and the use of technology.

The participants’ different proficiency levels in English proved to be an issue for some. A few participants compared their English comprehension and performance with others and said they felt they were falling behind. The rhythm of the sessions was also mentioned, with some participants saying that the classes went too quickly. Future research should thus take into account participants’ proficiency in the TL and adapt the course’s pace accordingly. Moreover, three of the five participants who did not attend final sessions were beginners in English. Participants’ limited experience with English could have been a demotivating factor for those with lower proficiency in English.

In contrast, participants who had already studied English seemed more comfortable with the intervention’s pace and organization. Indeed, adults with second-language experience show a learning advantage over those who are learning a language for the first time (Hansen et al., 2002). Future studies should thus give special attention to beginners, providing them with individual attention and a slow, steady pace.

The use of audiovisual tools was not mentioned by all of the participants, but three participants specifically mentioned the materials. The lack of comments on the program’s audiovisual content was perhaps due to the fact that during the interviews the psychologists did not directly inquire about the videos. However, much was said about the use of tablets.

This study used a method that integrated technology with traditional one-on-one instruction. This proved an extra challenge for some, while facilitating learning for others. The multimedia approach seemed to be quite useful for teaching and stimulating participants. In addition, having the participants use the tablets to look up words encouraged their involvement by providing them with the tools to translate on their own. However, only two participants mentioned using online English programs at home. Although some language-learning websites were introduced, the participants were not fully trained to use them. More computer training might have encouraged participants to study at home and provide continuity beyond the end of the program.

### Language Learning as a Social Activity

All of the participants mentioned an absence of social bonding with fellow participants, and some regretted not being able to form stronger social ties with one another. This could also be observed in the lack of significant improvement in the results from the UCLA questionnaire. Differences in English proficiency among participants may have hindered the creation of social bonds. One participant remarked that she thought some people did not belong in the group because of their advanced English skills. On the other hand, another participant mentioned that differences in English fluency could have encouraged participants to ask one another for help and thus foster social interaction. Perhaps the issue of generation also contributed to the lack of social ties, as one participant noted that those belonging to their generation did not make friends easily.

### The Significance of Language Learning

Nonetheless, learning activities have been shown to help older adults develop coping strategies and self-confidence (Boulton-Lewis, 2010). The study of language is a unique discipline, notably in regards to its social and subjective implications. Dörnyei (2005) writes that language concerns one’s social identity; therefore, learning a foreign language could change one’s self-image, facilitate the use of new behaviors and customs, and greatly impact the learner’s social being. Foreign-language learning could also affect psychological mechanisms, as language has been said to structure the unconscious itself (Lacan, 1966).

### Language Learning as Cognitive Stimulation

Pre- and post-intervention scores of the MoCA did not significantly differ. This is probably due to our study’s small sample size, as well as participants’ generally high cognitive level. These results could also imply that our intervention played a role in the maintenance of participants’ cognitive level. Although research has focused on early bilingualism’s contribution to cognitive reserve, it has been shown that even those who acquire a language after childhood have a cognitive advantage over monolinguals (Vega-Mendoza et al., 2015). For instance, compared to their monolingual peers, late bilinguals have demonstrated higher scores in tests of auditory attention span (Bak et al., 2014). In addition, differences in executive functioning can be attributed to second-language proficiency, and brain anatomy has been shown to change as language skills advance, even during the earliest stages of second-language acquisition (Mechelli et al., 2004; Osterhout et al., 2008).

Foreign-language acquisition has been shown to demonstrate structural neuroplasticity in children, adults, and older adults, even after short-term language training (Hosoda et al., 2013; Li et al., 2014; Pliatsikas et al., 2015). Learning a foreign language later in life could thus strengthen cognitive functioning in older adults.

Furthermore, with conversation training, recovery of a second language has been demonstrated in patients who have Alzheimer’s (Nold, 2005). With an adapted program and sufficient participant motivation, could this type of second-language learning program be appropriate for those with cognitive impairment? Further studies are needed to test the feasibility of such a program for those with MCI and dementia.

### Limitations

Although the current findings present a useful starting point for the development of cognitively stimulating, language-learning programs for older adults, this exploratory qualitative study cannot be generalized to the rest of the population due to its small size. The older adults were recruited from a panel of individuals who may have been particularly open to research and technology, as some of them had been involved in other studies at Broca Hospital in the past. Therefore, their opinions may not be representative of the general older French population. Additionally, not all of the participants were available for interviews at the end of the intervention, which could have biased the results. As for the structure of the interviews, the questions may have been too narrow in scope, and perhaps did not allow for participants to fully express their thoughts on the program. As this study is primarily qualitative, quantitative measures were used only for cognitive level and perceptions of loneliness. Furthermore, different proficiency levels in English were not controlled for, as beginner, intermediate, and advanced participants were included in the same group. English acquisition measures were also absent, and therefore it is unclear how much was genuinely learned by participants. In future studies, more thorough cognitive and psychological testing should be carried out, including executive functioning measures at pre- and post-intervention, as well as language acquisition tests.

## Conclusion

In our study we developed a technology-integrated, foreign-language program for seniors. We found that this program was feasible for a cognitively normal French senior group. In the future, it would be beneficial to have different groups for different language levels and adapt the program according to participants’ interests and basic needs. Further research should explore the quantitative cognitive effects of learning a second language in later life.

## Ethics Statement

This study was carried out in accordance with the recommendations of the ‘Commission Nationale de l’Informatique et des Libertés’ with written informed consent from all subjects. All subjects gave written informed consent in accordance with the Declaration of Helsinki. The protocol was approved by the ‘Commission Nationale de l’Informatique et des Libertés.’

## Author Contributions

A-SR supervised the project from the start, led focus groups throughout the study, read and analyzed the qualitative data, and greatly contributed to the writing and proofing of the manuscript. LD and SD were present during all of the sessions of the study in helping facilitate the use of tablets by participants. They were key to the development and execution of the study, conducting initial and post-evaluation interviews with participants, as well as analyzing the results and proofreading the manuscript. JB read and analyzed all the transcribed interviews. MP was present from the beginning of the project’s inception, attended important focus group meetings and contributed to the writing and proof-reading of the article. Y-HW contributed to focus group meetings, discussing the development and results of the program, and aided in the writing and proof-reading of the manuscript. VC guided CW in the project design with her expertise in qualitative research, she also helped to write and proofread the manuscript. CW came up with the idea of the program, and with the contribution of all the authors, developed and executed the program. She carried out post-intervention interviews, transcribed and analyzed them, and is the main author of the manuscript.

## Conflict of Interest Statement

The authors declare that the research was conducted in the absence of any commercial or financial relationships that could be construed as a potential conflict of interest.
